# A Role for Methyl-CpG Binding Domain Protein 2 in the Modulation of the Estrogen Response of *pS2*/*TFF1* Gene

**DOI:** 10.1371/journal.pone.0009665

**Published:** 2010-03-12

**Authors:** Amandine Chatagnon, Esteban Ballestar, Manel Esteller, Robert Dante

**Affiliations:** 1 INSERM, U590, Lyon, France; 2 Cancer Epigenetics and Biology Programme (PEBC), Catalan Institute of Oncology (ICO-IDIBELL), L'Hospitalet de Llobregat, Barcelona, Spain; Ludwig-Maximilians-Universität München, Germany

## Abstract

**Background:**

In human Estrogen Receptor α (ERα)-positive breast cancers, *5*′ end dense methylation of the estrogen-regulated *pS2/TFF1* gene correlates with its transcriptional inhibition. However, in some ERα-rich biopsies, *pS2* expression is observed despite the methylation of its TATA-box region. Herein, we investigated the methylation-dependent mechanism of *pS2* regulation.

**Methodology/Principal Findings:**

We observed interplay between Methyl-CpG Binding Domain protein 2 (MBD2) transcriptional repressor and ERα transactivator: (i) the *pS2* gene is poised for transcription upon demethylation limited to the enhancer region containing the estrogen responsive element (ERE); (ii) MBD2-binding sites overlapped with the methylation status of the *pS2* 5′ end; (iii) MBD2 depletion elevated *pS2* expression and ectopic expression of ERα partially overcame the inhibitory effect of MBD2 when the ERE is unmethylated. Furthermore, serial chromatin immunoprecipitation assays indicated that MBD2 and ERα could simultaneously occupy the same *pS2* DNA molecule; (iv) concomitant ectopic *ER*α expression and MBD2 depletion resulted in synergistic transcriptional stimulation, while the *pS2* promoter remains methylated.

**Conclusions/Significance:**

MBD2 and ERα drive opposite effects on *pS2* expression, which are associated with specific steady state levels of histone H3 acetylation and methylation marks. Thus, epigenetic silencing of *pS2* could be dependent on balance of the relative intracellular concentrations of ERα and MBD2.

## Introduction

Global loss of DNA methylation and localized CpG island hypermethylation is a common characteristic of cancer cells [1]–[3], leading respectively to aberrant ectopic gene activation or inversely to gene silencing. The *pS2* gene (also called *TFF1*) has been identified by differential screening of a cDNA library from the human breast cancer cell line MCF7 [4]. In this cell line, its transcription is directly controlled by estrogens [5] and an estrogen responsive element (ERE) has been identified at nt positions −405 to −393, from transcription start site [5]. In breast tumors, expression of the *pS2* gene is correlated with the presence of estrogen receptors (ER), and it had been suggested that *pS2* expression increases cell proliferation and tumor cell survival [6], [7]. Analysis of breast cancer biopsies or microdissected cells from formalin-fixed breast tissues has shown that *pS2* is hypomethylated in sub-classes of breast cancers [8], [9].

We have previously shown [8] that the hypomethylation of the CCGG site close to the *pS2* ERE correlates with its expression in human breast cancer biopsies. Southern blots performed with methylation sensitive enzymes and bisulphite sequencing have indicated that the breast tumors analyzed exhibited different DNA methylation patterns at the 5′ end of *pS2* [8]. Biopsies can display either methylated, unmethylated and partially methylated 5′ end *pS2* sequences at CpGs analyzed (nt positions −84 to +16) [8]. These observations prompted us to investigate the methylation-linked mechanisms of *pS2* gene repression and the potential involvement of DNA methylation in its response to estrogen stimulation.

In mammals, mechanisms implicated in the generation of a repressive state of chromatin associated with methylated DNA sequences have been investigated for over 20 years [10]–[13]. Pioneering studies led to the discovery of the Methyl-CpG binding domain (MBD) proteins family [14], which mediate DNA methylation-dependent gene silencing. The five *bona fide* MBD proteins, MeCP2, MBD1, MBD2, MBD3, and MBD4, share a canonical MBD. Biochemical and genetic analyses of these proteins have provided evidence of a direct link between DNA methylation and repressive chromatin architecture. MeCP2, MBD1 and MBD2 proteins bind to methylated DNA and recruit different histone deacetylase (HDAC)- and histone methyltransferase (HMT)-containing complexes that control chromatin compaction and gene silencing [15]–[17]. Mammalian MBD3, which lacks a functional MBD, does not recognize methylated DNA but is part of the histone deacetylase and chromatin remodeling Mi2/NuRD complex [18]–[20]. The last member of this protein family, MBD4, is a thymine glycosylase primarily involved in DNA repair [21].

The involvement of MBD proteins in gene imprinting [22], X inactivation [23], and transcriptional silencing of genes possessing hypermethylated CpG islands in cancer cells [2], [3] is now well documented. However, in contrast to the situation observed for DNMT-deficient mice, which either fail to develop or else die shortly after birth [24], the loss of MBD proteins, with the exception of MBD3, does not result in dramatic phenotypes [17], suggesting that MBD proteins deficiency causes subtle gene-expression changes.

Although the involvement of MBD proteins in gene silencing is well established, new facets regarding the links between DNA methylation, MBD proteins and gene transcription are emerging. For instance, it has been reported that DNA methylation in the body of genes can alter chromatin structure and reduce transcriptional elongation [25]. In parallel with the above findings, we have shown that the association of MBD2 with a methylated CpG island located downstream of the promoter region reduces the transcription of *NBR2* gene [26].

The density of methylation seems to be an important parameter in the MBD proteins-dependent repression. Several years ago, using *in vitro* methylated plasmids, it had been shown that the density of methylated CpG and promoter strength modulate transcriptional repression mediated by MeCP1 complexes containing MBD2 [27]. Furthermore, analysis of the transcriptional activity of patch-methylated plasmids microinjected into *Xenopus* oocytes has suggested a competition between transactivators and MBD proteins for the establishment of an open conformation [28]. All together, these data suggest that transcriptional repression mediated by DNA methylation is a consequence of a cross-talk between methylated CpG density, MBD proteins and transactivators.

To further explore the relative roles of methylated CpG patterns and the competition between transactivators and MBD proteins to influence or modulate gene transcription, we here investigate the expression of the estrogen-regulated *pS2/TFF1* (*Trefoil Factor 1*) gene [29] in cell lines exhibiting different DNA methylation patterns at its 5′ end, unmethylated, regionally methylated, and fully methylated. In these cell lines, it was possible to manipulate artificially the Estrogen Receptor-α (ERα), the natural transactivator of *pS2*, and MBD protein levels, and therefore, use them to determine the contribution of these proteins to *pS2* expression.

## Results

### Correlation between methylation patterns and *pS2* transcriptional repression

Expression of *pS2* is driven by a complex promoter containing a promoter/enhancer region responsive to estrogens, EGF, a phorbol ester tumor promoter, c-Ha-ras oncoprotein, and c-jun protein [33]. Specifically, the 5′ end of *pS2* possesses an estrogen-responsive element (ERE), conferring potential estrogen inducibility. In the ERα-rich MCF7 cell line, it has been shown [34] that proteins present on the *pS2* promoter in the absence of estradiol (E_2_) include basal transcriptional factors, active polymerase II, certain HATs and HMTs. This basal transcriptional activity implies steroid-independent expression of *pS2*. Moreover, the 5′ end of *pS2* (nt positions −464 to +314) is included in a CpG-poor region, G + C  = 0.54%, CpG observed / CpG expected  = 0.35. A correlation between the methylation status of the *pS2* promoter region and its expression has been observed in human tissues and the breast cancer cells lines [8], [35]. Experimental evidences are also in favour of a role of DNA methylation in the repression of *pS2* transcription. In the ERα-rich MCF7 cells, *pS2* is unmethylated and transcriptionally active while in the ERα-negative MDA MB231 cell line the 5′ end of *pS2* (nt positions −665 to +17) is fully methylated and the *pS2* gene is silenced, [35]. We have extended this analysis to the down stream region of *pS2* promoter, since it had been suggested that DNA methylation of the regions adjacent to a promoter region may affect transcription [26], [36], [37].

**Figure 1:**
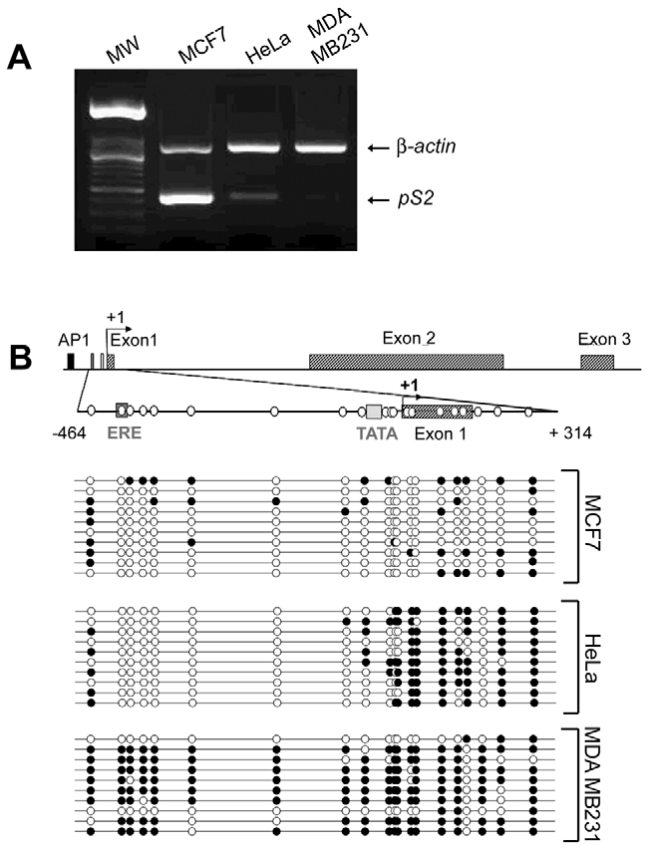
*pS2* gene expression and DNA methylation patterns in MCF7, HeLa and MDA MB231 cells. (**A**) The expression of endogenous *pS2* gene in MCF7, HeLa and MDA MB231 cell lines. *pS2* mRNA levels were monitored by relative RT-PCR. Briefly, *pS2* transcripts were simultaneously amplified with *β-actin* transcripts as a loading control and expression standard. (**B**) Methylation patterns at CpG sites of *pS2* 5′ flanking sequence from nt positions −464 to +314 in MCF7, HeLa, and MDA MB231 cell lines. A schematic representation of the human *pS2* gene is shown. The transcription start site is indicated by a black arrow. Black box, AP1 site; dark-grey box, Estrogen-Responsive Element (ERE); light-grey box, TATA-box; hatched boxes, *pS2* exons. The studied region (from nt positions −464 to +314) is presented on an expanded scale. This region contains 20 CpG sites, represented by white circles. The bisulphite-sequencing status of this 5′ *pS2* region in MCF7, HeLa and MDA MB231 cells (number of analyzed clones, n = 10) is represented. Each line corresponds to a single DNA template molecule. Black and open circles represent methylated and unmethylated CpGs, respectively.

The methylation status of the 5′ end of *pS2*, from nt positions −464 to +294, was investigated in MCF7 cells expressing *pS2* at a high level and MDA MB231 a *pS2* negative cell line (Figure 1A). Bisulphite conversion of DNA and sequencing of cloned PCR fragments, indicated that this region is unmethylated in MCF7 and fully methylated in MDA MB231 cells (Figure 1B). A screening failed to detect a human breast cancer cell lines exhibiting intermediate methylation patterns similar to that observed in breast cancer biopsies by Southern blot experiments [8]. Nevertheless, some of these patterns, associated with low level of *pS2* transcripts (Figure 1A), are very similar to that observed in HeLa cells [8]. Thus, these cells were chosen for further analysis. HeLa cells exhibit an intermediate DNA methylation pattern, the CpGs spanning the −464 to −84 region, which includes the ERE are unmethylated, while the TATA-box region is methylated (Figure 1B). These data confirm the inverse correlation between *pS2* expression and the density of the methylation of its 5′end, suggesting that the methylation patterns around the transcription start site impact in the activity of *pS2* promoter in these cells.

### Specific binding of MBD2 to the methylated *pS2* promoter

Among the proteins involved in the methylation-dependent repression of transcription, MBD proteins seem to play a major role. Therefore, we assessed the presence of MBD proteins on *pS2* promoter by chromatin immunoprecipitation (ChIP) assays using antibodies directed against MBD1, MBD2 and MeCP2.

**Figure 2:**
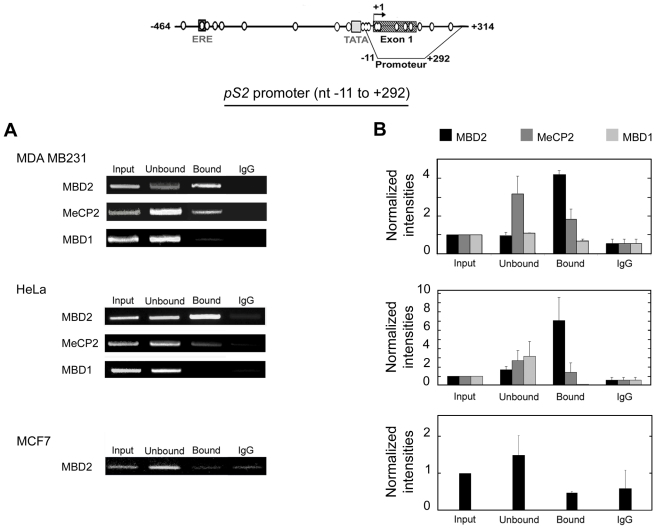
Specific association of MBD2 to the methylated promoter region of *pS2* gene. Detail of the *pS2* gene region analyzed (from nt positions −464 to +314). CpG sites are represented by circles. The black line represents the position of the fragment amplified by dose-dependant and quantitative PCR after ChIP. MBD proteins binding to the methylated region of the *pS2* promoter (from nt positions −11 to +292) was analyzed by ChIP in MDA MB231, HeLa and MCF7 cells. Cross-linked chromatin was immunoprecipitated using rabbit polyclonal anti-MBD2, anti-MeCP2 and anti-MBD1 antibodies. Purified DNAs from the input, unbound, bound or IgG fractions were quantified and an equal quantity of each fraction (0.5 ng) of this DNA was amplified by dose-dependent (**A**) or quantitative (**B**) PCR. (**A**) Representative experiments of MBD occupancy in the *pS2* promoter are shown. (**B**) Relative amounts of immunoprecipitaded *pS2* promoter to the input fraction measured by quantitative PCR. Each bar represents the mean ± standard deviation of at least three independent experiments.

Representative experiments from at least three independent assays for each antibody are shown in Figure 2A. As a control, the fractions immunoprecipitated with a non-MBD protein-specific antibody (anti-mouse IgG) were also analyzed. In order to determine *pS2* DNA fragment enrichment in MBD immunoprecipitated fractions, a dose-dependent and quantitative (Q-PCR) amplifications (Figure 2A, B) using an equal quantity of DNA (0.5 ng) per PCR assay, were performed with each fraction obtained from the ChIP procedure. We focused this analysis on the *pS2* methylated promoter region, shared by HeLa and MDA MB231 cells (nt positions −11 to +292).

In the *pS2*-methylated cells, MDA MB231 and HeLa, when antibodies against MBD2 were used, the amount of *pS2* promoter per ng of total DNA in immunoprecipitated fraction (Figure 2A, “bound”) was greater than in the input, or in the non-retained fractions (Figure 2A, “input”, “unbound”, and “IgG”), indicating that this methylated region is immunoprecipitated by anti-MBD2 antibodies. In contrast, ChIP assays with anti-MeCP2 or anti-MBD1 antibodies led to a depletion of this DNA segment in the bound fractions (Figure 2A). Western blot analysis, using antibodies directed against MeCP2 and MBD1, produced a signal of the expected sizes [14], ∼85 kDa and ∼70 kDa, respectively (data not shown), indicating that both proteins are expressed in MDA MB231 and HeLa cells. As an additional control of MBD2-ChIP assays, we also amplified the −11 to +292 *pS2* region from MCF7-chromatin immunoprecipitated by anti-MBD2 antibodies (Figure 2A). As expected, no enrichment was observed in the bound fraction, since this region was unmethylated in the MCF7 cell line (Figure 2A). Taken together, these data strongly suggest that MBD2 binds selectively and specifically the methylated region of this promoter.

### High resolution MBD2 binding profiles analysis of *pS2* promoter indicates that MBD2 specifically binds the methylated *pS2* promoter region and does not spread to the umethylated ERE in HeLa cells

In HeLa cells, the bimodal methylation status of the *pS2* 5′ end suggests that only some regions would actually be bound by MBD2. The unmethylated region containing the ERE (nt positions −405 to −393) is very close to the methylated TATA-box region (beginning at nt -9). ChIP experiments are not appropriate to discriminate between these two regions, as they are below the limit of resolution of the assay. Standard sonication of crosslinked chromatin leads to 300–500 bp DNA fragments and attempts to reduce its length (100–200 bp) resulted in a loss of efficiency in immunoprecipitation. Therefore, to precisely map the MBD2 binding sites we used a high-resolution method based on a ChIP-on-chip approach (Chatagnon *et al.*, manuscript in preparation). DNAs obtained from HeLa cell chromatin immunoprecipitated by anti-MBD2 antibodies were hybridized on an Affymetrix Human Promoter 1.0R Array (ChIP-on-chip).

**Figure 3:**
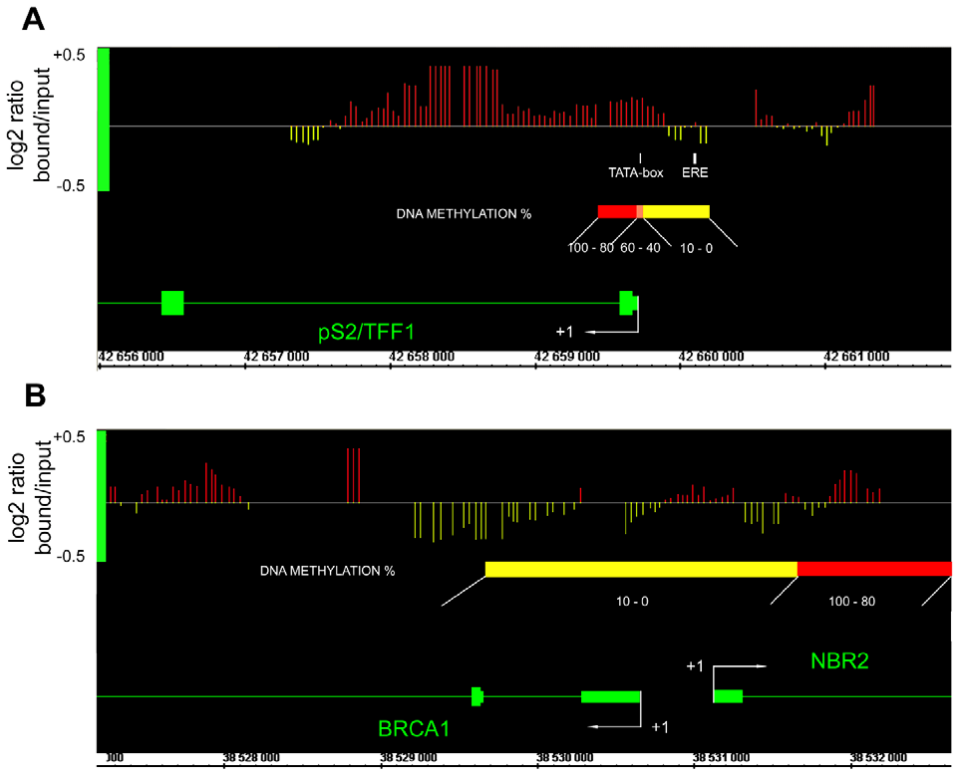
ChIP-on-chip analysis of MBD2-binding sites on *pS2* 5′ end regionally methylated in HeLa cells. (**A**) Array peaks on *pS2* 5′ end of MBD2 log2 signal ratio (MBD2 / Input) values are shown below the Affymetrix' Integrated Genome Browser (IGB) window. Red bars, MBD2 binding sites; yellow bars, MBD2 free sites. Genes are transcribed from right to left. *pS2* methylation status from nt positions −464 to +314 is shown by a diagram. “*pS2* ERE fragment” and “*pS2* promoter fragment” analyzed by PCR following ChIP are represented by a white box. (**B**) *BRCA1* 5′ end viewed as a MBD2 free control.

ChIP-on-chip experiments indicated that MBD2 associated specifically the region containing the methylated *pS2* TATA-box, where a strong positive signal (red bars) is observed, while the region containing the unmethylated ERE is devoid of MBD2. Thus, the positive signals for MBD2 binding parallels the methylation status of the *pS2* 5′ end and indicates that MBD2 does not spread outside the methylated region on *pS2* promoter (Figure 3A). As a control, results obtained for a previously identified MBD2 free promoter [26], *BRCA1*, are also shown on Figure 3B. Consistent with previous findings, no MBD2 positive signal was observed in the region spanning the nucleotides −1000 to +1000 of *BRCA1* (Figure 3B).

### MBD2 acts as a methylation-dependant transcriptional repressor of *pS2* transcription

The correlation between levels of *pS2* expression and the presence of MBD2 in the TATA-box region argues in favour of a repressive effect of MBD2 on *pS2* transcription. To examine this further, we depleted cells from MBD2 by transient transfection of siRNA targeted *MBD2* transcripts. Quantitative competitive RT-PCR assays indicated a significant reduction of the *MBD2* mRNA level (by 87–93%) (Supplemental Figure S1A). Moreover, in HeLa cells, western blot analysis also revealed a dramatically lower abundance of MBD2 proteins in the MBD2 siRNA-treated cells compared with control cells (Supplemental Figure S1B). Furthermore, the expression of MeCP2 and MBD1 was not different in *MBD2* knockdown HeLa cells than in wild-type or mock-treated cells (Supplemental Figure S1B).

**Figure 4:**
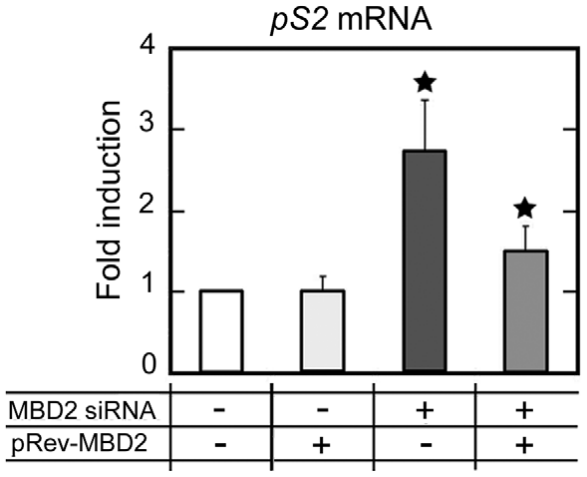
MBD2 specifically and directly represses *pS2* transcription. Real time RT-PCR analysis of *pS2* transcripts in HeLa and *MBD2*-depleted HeLa cells (HeLa cells pretreated for 72 h with MBD2 siRNA) transfected with an MBD2 vector expressing a transcript resistant to RNAi (pRev-MBD2 vector) or with an empty basic vector pGL3. Transcriptional expression of *pS2* was analyzed 24 h after transfection. The fold change of *pS2* expression was calculated from the relative *pS2* mRNA in pRev-MBD2-transfected cells compared to pGL3-transfected cells. Values are presented as the mean ± standard deviation of at least three independent transfection experiments. A significant difference between the two cell groups is represented by an asterisk * (P<0.05).

In the *pS2*-fully methylated cells MDA MB231, MBD2 depletion (about 90%), did not induce *pS2* expression (Supplemental Figure S1C). However, in MBD2 siRNA-treated HeLa cells, *pS2* expression is stimulated approximately 3-fold (Figure 4), while the methylation level of *pS2* TATA box region and ERα expression remain unaffected (data not shown). In MBD2 siRNA-treated HeLa cells, transient expression of an *Mbd2* cDNA, refractory to siRNA-mediated decay [26], shifted down the *pS2* mRNA level (Figure 4). Additional dose of Mbd2 in HeLa cells, containing normal levels of MBD2 proteins, did not affect *pS2* expression (Figure 4). Thus, the amount of MBD2 protein is not a limiting factor in the transcriptional repression of *pS2* in these cells. In MCF7 cells, quantitative RT-PCR showed that the level of *pS2* expression is unaffected by MBD2 depletion, indicating that MBD2 siRNA did not elevate *pS2* expression by an off-target effect since *pS2* is not bound by MBD2 in these cells (Supplemental Figure S1C).

### ERα only bound *pS2 ERE* when unmethylated in cell lines

To determine whether ERα can be recruited on the *pS2 ERE* sequence when this region or the adjacent region is methylated, we artificially manipulated the level of ERα in MDA MB231 and HeLa cells, which are deficient in this protein.

**Figure 5:**
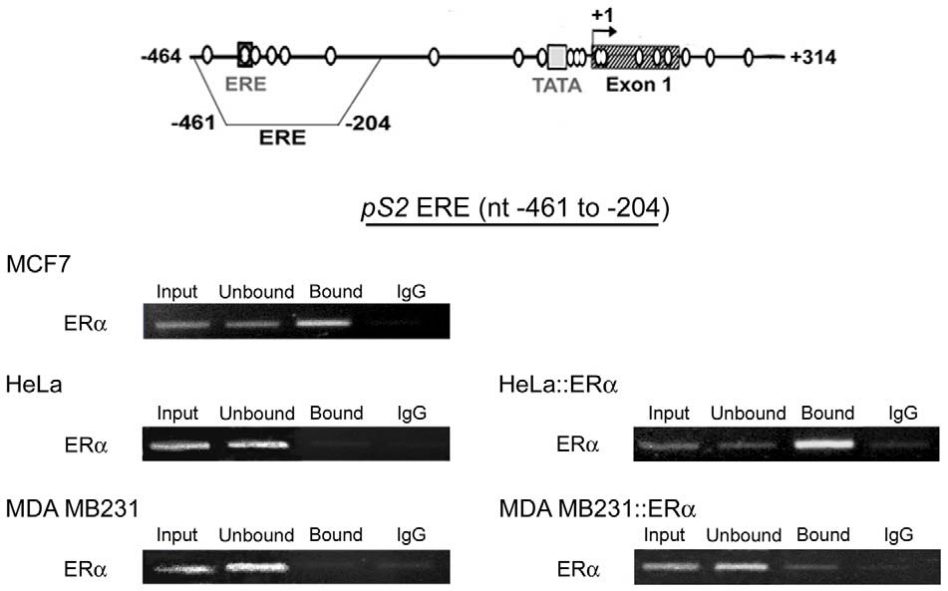
ERα only associates hypomethylated ERE region of *pS2*. Representative experiments of ERα ChIP assays in ERα-rich MCF7 cells, in ERα-negative HeLa and MDA MB231 cells, and in HeLa and MDA MB231 expressing the vector HEG0 encoding ERα (HeLa::ERα, and MDA MB231::ERα). ChIP assays were performed as described in Figure 2. The position of the “*pS2* ERE fragment” analyzed by PCR are represented on the *pS2* 5′ end schema.

ChIP assays, using anti-ERα antibodies, were performed from, HeLa and MDA MB231 cells transiently transfected with the vector HEG0 encoding ERα. ERα-rich MCF7 cells were used as a positive control and untransfected HeLa and MDA MB231 cells as negative controls. As expected, ChIP assays performed from MCF7 chromatin indicated that the amount of *pS2* DNA per ng of total DNA in immunoprecipitated fraction (Figure 5, “bound”) is higher (about 8-fold, Q-PCR assays) than in input, or non-retained fractions (Figure 5, “input”, “unbound”, and “IgG”) while no enrichment in *pS2* sequence was observed in immunoprecipitated fraction obtained from untransfected HeLa and MDA MB231 cells (Figure 5). All together, these data indicate that anti-ERα antibodies specifically precipitated chromatin bound by ERα.

In HeLa cells, transient expression of the vector HEG0 coding for ERα, leads to enrichment in *pS2* sequence in the immunoprecipitated fraction (Figure 5). This enrichment (about 10 fold, Q-PCR assays) was comparable to that observed in MCF7 cells, indicating that ERα is efficiently recruited on the *pS2* ERE site in HeLa cells. In contrast, in MDA MB231 cells, despite ectopic expression of ERα, *pS2* sequence was not selectively immunoprecipitated by the anti-ERα antibodies (Figure 5). Thus, ERα does not bind the methylated *pS2* ERE in MDA MB231 cells, suggesting that full DNA methylation induces chromatin changes that prevent ERα binding.

### Ectopic expression of *ERα* enhances *pS2* gene expression only when its 5′ end region is partially methylated, in ERα negative cells

The ERE of the *pS2* promoter can act as a strong enhancer in the presence of E_2_ and ERα [29], [33]. To analyze the potential antagonistic activity of MBD2 and ERα on *pS2*, ectopic expression of ERα was induced in MDA MB231 and HeLa cells.

**Figure 6:**
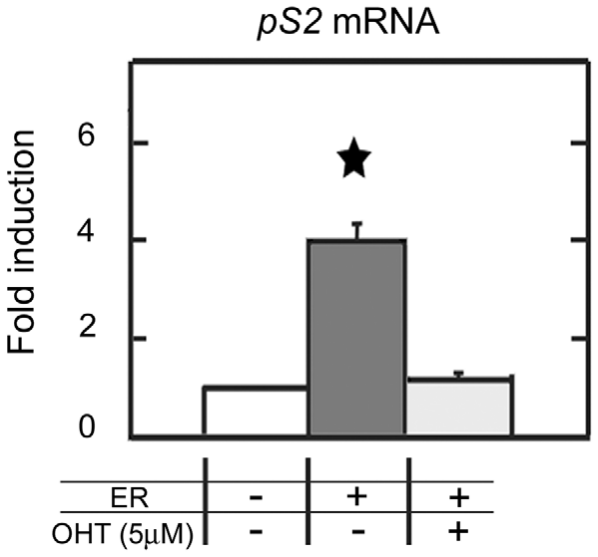
ERα stimulates *pS2* transcription in HeLa cells. Real time PCR quantification of *pS2* mRNA in HeLa cells ectopically expressing ERα. HeLa cells were transfected with the vector HEG0 coding for ERα and *pS2* expression was monitored 24h after. To investigate the estrogen dependence of *pS2* expression, cells were exposed to 5 µM of antiestrogen (4-hydroxytamoxifen or OHT). Bar chart show the fold change of *pS2* expression calculated from the relative *pS2* mRNA in HEG0-transfected cells compared to pSG5, empty vector-transfected cells. Each bar represents the mean ± standard deviation of three analyses. A significant difference between the two cell groups is represented by an asterisk * (P<0.05).

Transient expression of ERα elevated *pS2* expression by a 4-fold (Figure 6), in HeLa cells. This stimulation by ERα of *pS2* transcription did not affect the methylation status of HeLa *pS2* promoter (data not shown). ERα stimulation of *pS2* expression was fully reversed by antiestrogen (4-hydroxytamoxifen, OHT) treatments (Figure 6), while the basal level of *pS2* expression was conserved. These results indicate the existence of estrogen-dependent transactivation of *pS2* in HeLa cells. As expected, *pS2* expression was not induced in MDA MB231 cells (data not shown), since ERα did not bound *pS2*-ERE sequence in this cell line (Figure 5). These results suggest that demethylation of ERE region allows the estrogen response of *pS2* in HeLa cells.

### MBD2 is not displaced from the *pS2*-methylated promoter region by ERα transactivation, in HeLa cell*s*

**Figure 7:**
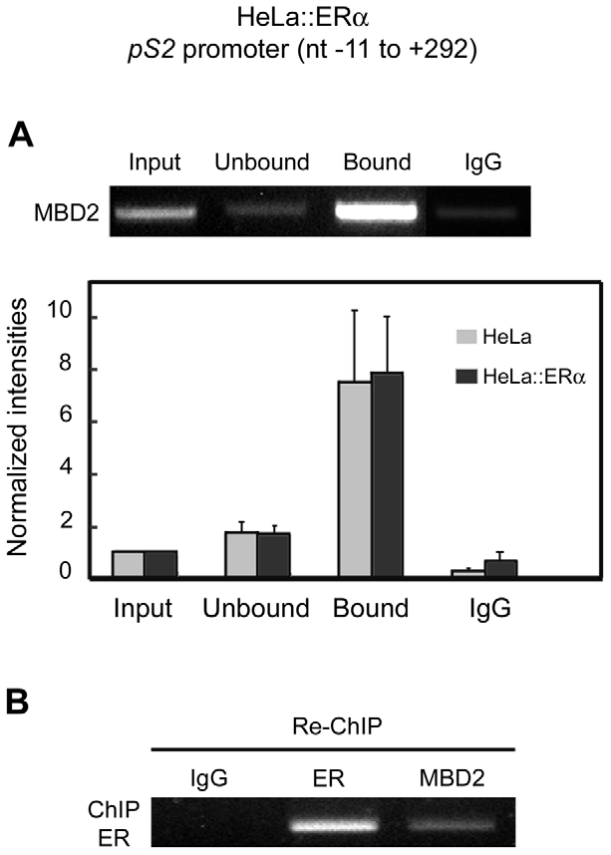
The transactivators ERα and the methylation-dependant repressor MBD2 can simultaneously bound the *pS2* promoter in HeLa cells. (**A**) MBD2 ChIP assays in HeLa cells expressing ERα (HeLa::ERα). Relative amounts of immunoprecipitaded *pS2* promoter measured by quantitative PCR from HeLa or HeLa::ERα cells. Each bar represents the mean ± standard deviation of at least three independent experiments. (**B**) Serial ERα-MBD2 ChIP assays to *pS2* promoter. Chromatin prepared from HeLa cells transfected with a human ERα expression vector was subjected to the ChIP procedure with the anti-ERα antibody, and again immunoprecipitated using antibodies as indicated at the top of the figure (Non-specific antibody, IgG; anti-ERα antibody, ER; anti-MBD2 antibody, MBD2).

HeLa cells expressing the vector encoding ERα were used to identify the proteins bound to the 5′ end of *pS2*. Dose-dependent and quantitative PCR amplifications of each fraction obtained from the ChIP procedure were performed. These assays showed that MBD2 was still present on the methylated region of *pS2* promoter (Figure 7A), and the enrichment in the bound fraction was not modified by the presence of ERα on the ERE (Figure 7A). These results provide evidence that ERα can overcome, at least partially, the inhibitory effect of MBD2 binding to *pS2* promoter and imply that both proteins can occupy the 5′ end of the *pS2* gene.

To address this matter, we performed serial ChIP assays. A first round of immunoprecipitation was carried out with an anti-ERα antibody from HeLa cells transiently transfected with the vector HEG0 encoding ERα. Then, immunoprecipitated cross-linked DNA-protein complexes were isolated and subjected to reimmunoprecipitation using antibodies directed against MBD2. PCR amplification of *pS2* promoter region from the fraction reimmunoprecipitated with anti-MBD2 antibodies gave a positive signal (Figure 7B). In control reactions, as expected, no signal was detected with non-specific antibodies, while a positive signal was observed in the fraction reimmunoprecipitated with anti-ERα antibodies (Figure 7B). Thus, the binding of ERα on the ERE of *pS2* does not displace MBD2 from the methylated TATA-box region, since both proteins were present on the same DNA molecules.

### Synergic activity of MBD2 depletion and ectopic *ER*α expression on *pS2* transcription, in HeLa cells

**Figure 8:**
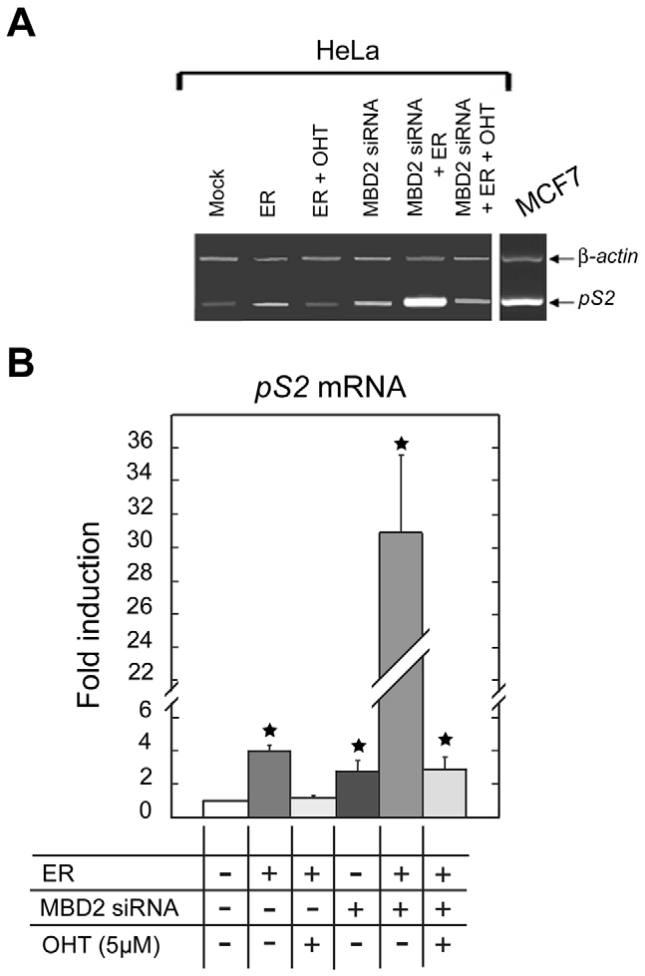
MBD2 modulates the estrogen response of *pS2* gene. (**A**) Transcriptional expression of *pS2* was analyzed using relative RT-PCR in HeLa cells expressing ERα and/or depleted in MBD2. Mock, mock-treated HeLa cells. ER, HeLa cells 24 h after transfection with a human ERα expression vector, HEG0. MBD2 siRNA, HeLa cells pretreated for 72 h with MBD2 siRNA and again for 24 h. MBD2 siRNA + ER, HeLa cells pretreated for 72 h with MBD2 siRNA, then cotransfected with MBD2 siRNA and HEG0 for 24 h. OHT, 24 h treatment with 4-hydroxytamoxifen. MCF7, MCF7 cells. (**B**) Bar chart showing the fold change of *pS2* expression in HeLa cells expressing ERα and/or depleted in MBD2. *pS2* transcripts were quantified by real time RT-PCR. The fold change was calculated from the relative *pS2* mRNA in treated compared to mock-treated cells. Each bar represents the mean ± standard deviation of three analyses. A significant difference between the two cell groups is represented by an asterisk * (P<0.05).

The opposite effects of MBD2 and ERα proteins on *pS2* expression suggest an antagonistic action between these two transcriptional regulators, in HeLa cells. To investigate this possibility, the concentrations of MBD2 and ERα proteins were artificially manipulated in these cells. After MBD2 depletion mediated by RNA interference, ectopic *ER*α expression resulted in a dramatic (approximately 31-fold) enhancement of *pS2* mRNA concentration, approaching the level to that observed in MCF7 (Figure 8A, B). Thus, *pS2* responses to ERα activation (4-fold increase) and MBD2 depletion (3-fold increase) are not additive and suggest a cross-talk between these two transcriptional regulators. Concomitant exposure to OHT knocked down *pS2* expression to the level observed in HeLa cells transfected by MBD2 siRNA alone, (about 3-fold) when compared with control HeLa cells (Figure 8A, B). It should be noted that *pS2* transcription cannot be induced by concomitant ectopic expression of ERα and MBD2 depletion when its 5′ end is fully methylated, as observed in MDA MB231 cells (data not shown). In cells exhibiting a bimodal methylation profile as HeLa cells, a synergic activation was observed. Thus, the binding of MBD2 to the methylated TATA-box of *pS2* reduces but does not abolish *pS2* response to ERα, suggesting that as a result of regional demethylation, *pS2* is poised for transcription.

### Post-translational modifications of histone H3 are associated with *pS2* expression induction

To get further insight on the mechanism involved in the opposite effect of MBD2 and ERα on *pS2* transcription, we investigated histone modifications in HeLa cells. It is well known that both proteins, MBD2 and ERα, regulate the transcription by the recruitment of chromatin remodeling complexes [15], [38] Importantly, ERα has been shown to interact with several coactivators with histone acetyltransferase activity (CBP, p300, p/CAF and the members of p160 family) [38], or histone demethylase (LSD1) [39]. Conversely, MBD2 recruits corepressors with histone deacetylase activity (Mi2/NurD) [17]. Histone H3 acetylation (H3Ac) and histone H3 lysine 9 (H3K9) trimethylation chromatin marks have been the subject of intense investigation during the past few years and appear to be associated with active and silent promoters, respectively.

**Figure 9:**
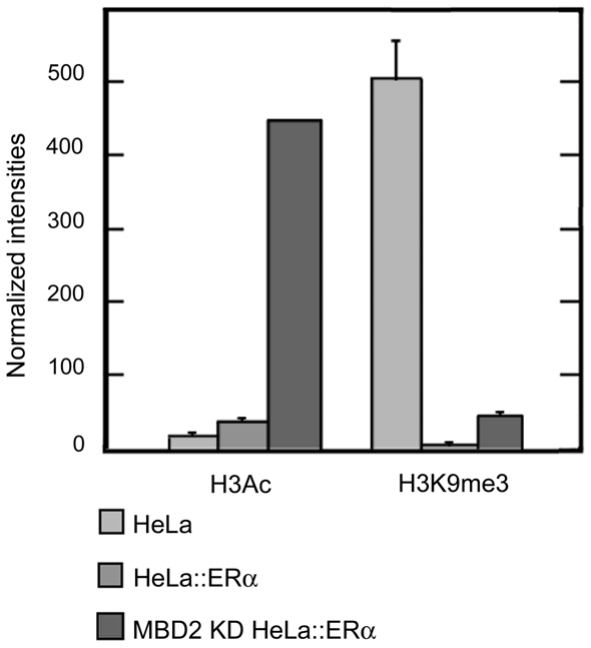
Histone H3 marks on *pS2* promoter in presence or absence of MBD2 and/or ERα. HeLa cells, wild type (HeLa), expressing ectopically ERα (HeLa::ERα), and depleted in MBD2 and expressing ectopically ERα (MBD2 KD HeLa::ERα), were subjected to ChIP analysis using anti-histone H3 acetylation (H3Ac) or an anti-histone H3 lysine 9 trimethylation (H3K9me3) antibodies. The *pS2* promoter was amplified by real-time PCR from an equal amount (0,5 ng) of total DNA immunoprecipitated by the different antibodies. Relative amounts of H3Ac or H3K9me3 marks were measured by comparing fractions immunoprecipitated by the anti*-*H3Ac or anti-H3K9me3 antibodies to fractions immunoprecipitated by the anti-histone H3 pan antibody. Each bar represents the mean ± standard deviation.

In our study, ChIP assays indicated that ERα *pS2* stimulation was associated with increase in histone H3 acetylation (∼2.5 fold) and enhanced the demethylation of H3K9 (∼500 fold) at *pS2* promoter, when compared with wild type HeLa cells (Figure 9). Moreover, in HeLa cells, the synergic activity of MBD2 depletion and ectopic ERα expression on *pS2* transcription led to a stronger induction of histone H3 acetylation (∼28 fold) at *pS2* promoter, while H3K9 methylation was still lowered (∼10 fold), (Figure 9). From these findings we conclude that the transcriptional MBD2 repressor and ERα transactivator co-participate to the regulation of *pS2* expression by mediating a balance between repressive histone H3 lysine 9 trimethylation and active histone H3 acetylation marks at *pS2* promoter. Thus, the repressive effect of MBD2 on the transactivation of *pS2* mediated by ERα is linked to histone modifications.

## Discussion

In cancer tissues and cell lines, transcriptional silencing associated with aberrant methylation of promoter regions is now regarded as an almost universal epigenetic marker of malignant transformation [1]–[3]. Since the first experiments showing that the MBD2 binds *in vivo* to the methylated regulatory regions of *p16* and *p14* and could thereby contribute to gene silencing in colon carcinoma cell lines [40], a body of evidence has accumulated concerning associations between MBD proteins and hypermethylated promoter regions [41]–[43]. In non-pathological situations, MBD proteins are also directly involved in the repression of imprinted genes and differentiation-dependent gene expression [17].

In unmethylated MCF7, methylated MDA MB231, and regionally methylated HeLa cells, we found that MBD2 binding profiles parallel the methylation patterns of the *pS2* 5′ end, while MeCP2 and MBD1 were not detected in this region. Furthermore in HeLa cells, high resolution mapping of MBD2 binding sites indicated that MBD2 only associated the methylated region close to the TATA-box, whereas the unmethylated region, including the *pS2* ERE, was not targeted by this repressor.

MBD2 depletion by siRNA targeting *MBD2* transcripts elevated *pS2* transcription by a factor of ∼3 in HeLa cells, while, in unmethylated cells (MCF7), *pS2* expression was not affected by MBD2-specific siRNA transfection suggesting a direct relationship between *pS2* repression and MBD2 binding.

Injection in *Xenopus laevis* oocytes of regionally methylated plasmids has shown that a few methylated cytosines can inhibit a flanking promoter but a threshold of modified sites is required to organize a stable, diffusible chromatin structure. From these data and results obtained with chemical inhibitor of histone deacetylase, these authors have proposed that a specialized chromatin structure, formed not only by MBD proteins but also by other structural and remodelling activities, is organized on the modified DNA, when the number of methylated sites is increased and reaches the threshold that leads to diffusion of gene silencing on the DNA fiber [28]. In agreement with this hypothesis, MBD2 depletion did not induce *pS2* transcription in MDA MB231 cells exhibiting fully methylated *pS2* 5′ end.

The present study also indicates that ERα only associates the unmethylated *pS2* ERE independently of the methylation status of the TATA-box region, since the same level of ERα was observed at the ERE region in MCF7 cells and in HeLa cells transiently transfected with the HEG0 vector encoding this receptor. In contrast, strong expression of ERα upon transient transfection with HEG0, did not lead to ERα binding in MDA MB231 cells, suggesting that full methylation of the 5′ end of *pS2* prevents its binding. Although a direct effect cannot be totally excluded, we have previously shown, using electrophoretic mobility assays, that methylated oligonucleotides containing the *pS2* ERE are efficiently recognized by ERα [44]. *In vivo* experiments also suggest an indirect effect of DNA methylation. In the HE5 cell line derived from MDA MB231 that expresses functional ERα, but not *pS2*, DNase I hypersensitive sites are not modified by ERα expression, while in the ERα and *pS2* positive MCF7 cells display hormono-dependent hypersensitive DNase sites at the *pS2* ERE region [45]. Thus, the methylation of TATA-box region does not seem to lead to diffusible alteration of chromatin structure, in HeLa cells.

As expected from the analysis of methylation and ERα binding sites profiles, ectopic expression of ERα in MDA MB231 cells did not stimulate *pS2* expression. In HeLa cells, ERα induction of *pS2* expression was observed suggesting that the demethylation of ERE region seems to be a prerequisite for estrogen-dependent *pS2* stimulation. Nevertheless, the level of *pS2* transcript remained relatively low when compared with that observed in E_2_ treated MCF7 cells. The present study indicates that high levels of ERα can at least partially overcome the transcriptional repression mediated by MBD2 without affecting the methylation status of the *pS2* promoter. Thus, the binding of MBD2 to the methylated TATA-box of *pS2* reduces but does not abolish *pS2* response to ERα.

The mapping of MBD2 binding sites at *pS2* locus indicated that MBD2 proteins lay downstream the initiation start site. The analysis of the 5′ region of the endothelin receptor B gene in human cell lines shows that extensive methylation closely downstream of the initiation site does not abolish gene expression [46]. However, the impact of intragenic methylation has been studied from transgenes methylated exclusively in a region downstream of the promoter, into a specific genomic site. This methylation pattern induces a close chromatin conformation and decrease transcription levels, suggesting that this epigenetic mark may reduces the efficiency RNA polymerase II elongation [25]. Thus, we cannot exclude that DNA methylation and MBD2 binding downstream *pS2* promoter region may impact elongation rate. However, we observed post-translational modifications of histone H3 (acetylation and demethylation) at the promoter region, when *pS2* transcription is induced by MBD2 siRNA and/or ERα expression, suggesting that MBD2 depletion influences chromatin conformation at *pS2* promoter.

Recently, it has been published that, upon E_2_ induction, synchronized human cell lines exhibit a cyclical methylation/demethylation of the *pS2* ERE region correlated with cyclical binding of transcriptional repressors/activators [47], [48], when the 5′ end of *pS2* is unmethylated [35], [48]. Although the maintenance of DNA methylation patterns begin to be well described, it will also be important to consider mechanisms that enable the removal of these marks to fully comprehend the dynamic behavior of DNA methylation, as suggested by recent reports [47], [48]. Nevertheless, the mechanisms and enzymatic activities that are responsible for DNA demethylation in mammals although potentially linked to DNA repair are controversial [47]–[53].

Transcriptional repression mediated by MBD proteins can be reversed by various mechanisms. In mouse and rat, Mecp2 is directly involved in a depolarization-controlled repression of the *brain-derived neurotrophic factor* (*Bdnf*) gene in neurons [54], [55]. This protein, associated with the corepressor Sin3a, binds to a CpG-poor methylated region of *Bdnf* promoters. Upon the initiation of Ca^++^ signaling, Mecp2 becomes phosphorylated and is liberated from the promoter as *Bdnf* is activated. Mbd2 is also able to repress CpG-poor methylated promoter. In Mbd2 null mice, ectopic expression of interleukin-4 (Il4) disrupts T-helper cell differentiation, suggesting that Mbd2 is a transcriptional repressor of Il4 in naïve T cells. Indeed, in naïve wild type T cells, overexpression of the transcription factor GATA-3, normally required for Il4 expression, displaces Mbd2 from the promoter and activates Il4 transcription [56].

The interplay between ERα and MBD2 in *pS2* transcription indicates that the partial reversion of transcriptional repression mediated by MBD2 does not necessary involve the displacement of this repressor from its CpG-poor promoter region. In the model studied, the amounts of *pS2* gene immunoprecipitated by anti-MBD2 antibodies was not affected by the binding of ERα. In addition, serial ChIP assays showed that *pS2* promoter region can be simultaneously bound by ERα and MBD2 proteins. Furthermore, the synergic effects of ectopic ERα expression and depletion of MBD2 on *pS2* transcription also suggest that both proteins do not compete for *pS2* promoter occupancy.

Data, obtained from experiments performed on cell lines, indicate that methylation-dependent repression of *pS2* expression is mediated by MBD2. Nevertheless, ERα binding can counteract the inhibitory effect of DNA methylation without displacing MBD2 proteins from the TATA-box region. Taken together these data might explain pS2 expression in some ERα-rich breast cancers despite the methylation of its TATA-box.

## Materials and Methods

### Cell culture

MDA MB231, MCF7, and HeLa cells were obtained from the American Type Culture Collection (ATCC, Rockville, MD). Cells were maintained in Dulbecco's modified Eagle's medium (DMEM) containing 1g/l glucose (Eagle, Sigma, L'isle d'Abeau, France) and supplemented with 10% of heat inactivated fetal bovine serum (Lonza, Vervier, Belgium) and grown at 37°C in a humidified 5% CO_2_ atmosphere.

Due to the poor efficiency of repeated siRNA treatments associated with vector transfections in estradiol depleted medium by charcoal extraction, all the experiments were performed in standard DMEM medium supplemented with-fetal bovine serum. To investigate the estrogen dependence of *pS2* expression, cells were exposed to estradiol or to 5 µM 4-hydroxytamoxifen (OHT).

### Sodium bisulphite modification

Sodium bisulphite reactions were carried out as previously described [30]. Two regions (nt positions −464 to +67, and nt positions +37 to +314 from the *pS2* transcription start site) within the *pS2* promoter gene were analyzed. PCR amplifications were accomplished in 100 µl using the HotStart *Taq* DNA polymerase Kit (Qiagen, Courtaboeuf, France) and 0.25 µM of the primers (Supplemental Table S1), after 15 min at 95°C for *Taq* polymerase activation and 35 cycles (30 s denaturation at 94°C, 1 min annealing at 52°C, and 1.5 min extension at 72°C). To determine accurately the proportion of methylated CpG, PCR products were cloned into a pGEM-T vector (Promega, Lyon, France) and 10 random clones from each sample were analyzed by automatic sequencing (Biofidal, Lyon, France).

### Chromatin Immunoprecipitation (ChIP) assay*s*

Nucleoprotein complexes were sonicated to reduce the length of DNA fragments to 300–600 bp, and ChIP assays were carried out as described previously [26]. Fithteen µl of two different polyclonal anti-MBD2 antibodies (kindly provided by Dr. P. Wade and Dr. E. Ballestar) or 20 µl of polyclonal anti-MBD1 (Abcam, Cambridge, UK), anti-MeCP2 (Upstate Biotechnology, Lake Placid, NY) antibodies, or 2.5 µg of polyclonal anti-ERα antibody (Santa Cruz Biotechnology, Inc, Santa Cruz, CA) or anti-mouse IgG (Dakocytomation, Trappes, France), were used for immunoprecipitation. DNA samples obtained from the input, unbound and bound fractions were quantified by densitometry using the VersaFluor™ Fluorometer (Biorad, Ivry, France) and RiboGreen reagent (Molecular Probes, Interchim, Montluçon, France). The amounts of DNA in IgG control fractions were at the limit of the fluorometric detection methods. Thus, PCR quantification of DNA fragments in IgG fractions was not accurate as in other fractions, since we have used very large parts of the “IgG fractions” when compared with the other fractions.

PCR assays were performed to assess the binding of the proteins to the *pS2* 5′ flanking sequence. Two regions were analyzed: “*pS2* ERE fragment” (from nt positions −461 to −204 from the *pS2* transcription start site) and “*pS2* promoter fragment” (from nt positions −11 to +292). We amplified, by dose-dependent and quantitative PCR (Q-PCR), equal amounts of total DNA (0.5 ng) from the input, unbound and bound fractions. HotStar *Taq* polymerase kit (Qiagen) and 0.4 µM of the primers (Supplemental Table S1) were used in classical PCR. After 15 min at 95°C for *Taq* polymerase activation and 37 cycles (30 s denaturation at 94°C, 1 min annealing at 53°C, and 1.5 min extension at 72°C) for the “*pS2* ERE fragment” or 36 cycles (30 s denaturation at 94°C, 1 min annealing at 58°C, and 1.5 min extension at 72°C) for the “*pS2* promoter fragment”, PCR products were analyzed on a 2% agarose gel containing 1 µg/ml ethidium bromide and were quantified by densitometry. Real-time PCR was carried out using LightCycler ® Fast Star DNA Master PLUS SYBR Green I System (Roche Molecular Biochemicals, Maylan, France) and 0.4 µM of the primers (Supplemental Table S1). Cycling parameters were 95°C for 10 min followed 45 cycles at 95°C for 10 s, 64°C for 5 s and 72°C for 10s.

### ChIP-on-chip

For ChIP-on-Chip analysis, the specific protein-DNA complexes were obtained from independent immunoprecipitations using two different polyclonal anti-MBD2 antibodies (kindly provided by Dr. P. Wade and Dr. E. Ballestar). The ChIP DNAs from the input and bound fractions were amplified, labelled and hybridized on microarrays by ProfileXpert service according to Affymetrix™ protocols. Briefly, the ChIP DNA was amplified by random PCR. Enrichment of MBD2-bound sites during the amplification procedure was assayed, by PCR amplification of *NBR2* [26] and *pS2* promoters, on each ChIP samples before and after amplification. The amplified DNAs were then labelled using the GeneChip® WT Double - Stranded DNA Terminal Labelling Kit and hybridized to the human tiling arrays (Human Promoter 1.0R Arrays), which were then washed and scanned. Raw data from the scans were analyzed using Affymetrix® Tiling Analysis Software (TAS) and the results were viewed in Affymetrix' Integrated Genome Browser (IGB) Software.

### Serial ChIP assays (ChIP re-ChIP)

The ERE and the TATA box regions of *pS2* are placed about 450 bp apart. In order to immunoprecipitate, from the same DNA fragments, the proteins bound to both regions, nucleoprotein complexes were sheared to reduce the length of DNA fragments to 500–1000 bp. In serial ChIP experiments, following primary immunoprecipitation, the cross-linked complexes were eluted from the immunoprecipitated fraction by incubation with elution buffer (1% SDS, 50 mM NaHCO_3_) at room temperature for 30 min, and then diluted 1:10 in ChIP dilution buffer (0.01% SDS; 1.1% Triton X-100, 1.2 mM EDTA, 16.7 mM Tris-HCl, pH 8.0, 167 mM NaCl) followed by reimmunoprecipitation with a second set of antibodies.

### Transient MBD2 siRNA knockdown and ERα vector transfections

siRNA duplexes for *MBD2* (sense: 5′-GGAGGAAGUGUACCGAAATT-3′; antisense: 5′-UUUUCGGAUCACUUCCUCCTT-3′) and non-specific siRNA control were obtained from Eurogentec (Eurogentec, Seraing, Belgium). HEG0, an expression vector coding for human ERα [31], and the empty vector pSG5, were provided by Prof. P. Chambon. MBD2 siRNA and ERα vector was transfected with Lipofectamine 2000 (Invitrogen, Carlsbad, CA) according to the manufacturer's instructions. Briefly, cells were seeded at 2×10^5^ cells per well in six-well plates, and grown to 50–60% confluence on the day of transfection. All transfections were done in Opti-MEM medium (Invitrogen) with 625 nM of MBD2 siRNA and 1 µg of ERα expression plasmid. Lipofectamine 2000 complexes were incubated for 4–5 hours. The medium was then removed and replaced with fresh medium. Cells were grown and harvested at various times after the transfection.

### Reverse-transcription-PCR analysis

Total RNA was extracted from the cell lines using the RNeasy Mini kit (Qiagen). After extraction, the integrity of total RNA was examined on a 1.2% agarose gel containing 1 µg/ml ethidium bromide and quantified by densitometry using a Fluor's fluorimeter and Quantity One software (Biorad, Ivry, France) by comparison with serial dilutions of a standard RNA (Roche, Molecular Biochemicals, Maylan, France).

*pS2* mRNA was detected by relative RT-PCR using primers described in Supplemental Table S1. Briefly, 0.1 µg of total RNA were amplified simultaneously for *β-actin* and *pS2* using the One Step RT-PCR kit (Qiagen). After 30 min incubation at 50°C, RT was inactivated by heating at 95°C for 15 min. PCR amplification was then performed under the following conditions: 30 cycles, 30 s denaturation at 94°C, 1 min annealing at 55°C and 1.5 min extension at 72°C. PCR products were analyzed on a 2% agarose gel and quantified. The ratio between *pS2* and *β-actin* signals was then determined.

Real-time RT-PCR were also carried out to quantify *pS2* mRNA using LightCycler® RNA Master SYBR Green I One-Step RT-PCR mix on a LightCycler® 2.0 system according to the manufacturer's instructions (Roche). *β-actin* mRNA was used as a reference. The primers sequences used for reverse-transcription-PCR are available in Supplemental Table S1.

*MBD2* mRNA was quantified by competitive quantitative RT-PCR as previously described [32].

## Supporting Information

Figure S1 MBD2 siRNA treatments, supplementary data. (A) MBD2 expression in MBD2 siRNA transfected cells. Bar chart representing the efficiency of MBD2 siRNA in HeLa, MCF7 and MDA MB231 In mock treated cells, the initial amount of MBD2 molecules / µg of total RNA was: 7.4 106±1.3×106, in HeLa cells; 5.8×106 ±1×106, in MDA MB231 cells and 3.2×106±0.6×106, in MCF7 cells. The efficiency of MBD2 siRNA was calculated from the MBD2 mRNA in treated cells compared with mock-treated cells. Each bar represents the mean ± standard deviation of, at least, three independent analyses. (B) MBD1, MBD2 and MeCP2 protein quantifications in HeLa cells expressing transient MBD2 siRNA. HeLa cells were pretreated for 72 h with MBD2 siRNA and again for 24 h. Mock-treated cells were transfected with a non-specific siRNA. Immunoblot analysis of MBD2, MBD1 and MeCP2 proteins in mock-treated and in MBD2 siRNA-treated HeLa cells. MBD2, MBD1 and MeCP2 proteins were probed using rabbit polyclonal antibodies. The same membrane was then stripped and probed using a mouse β-tubulin antibody as a loading control. (C) Bart chart showing the fold change of pS2 expression in MCF7 and MDA MB231 cells depleted in MBD2. pS2 transcripts were quantified by real-time RT-PCR. The fold change was calculated from the amount of pS2 mRNA in treated cells compared with mock-treated cells. Each bar represents the mean ± standard deviation of, at least, three independent analyses.
(0.52 MB TIF)

Table S1 List of primers.
(0.03 MB DOC)
