# Supplementary figures and images for: A Role for Methyl-CpG Binding Domain Protein 2 in the Modulation of the Estrogen Response of pS2/TFF1 Gene
Source: PLoS One. 2010 Mar 12;5(3):e9665. doi: 10.1371/journal.pone.0009665 (PMC2837351; doi:10.1371/journal.pone.0009665)

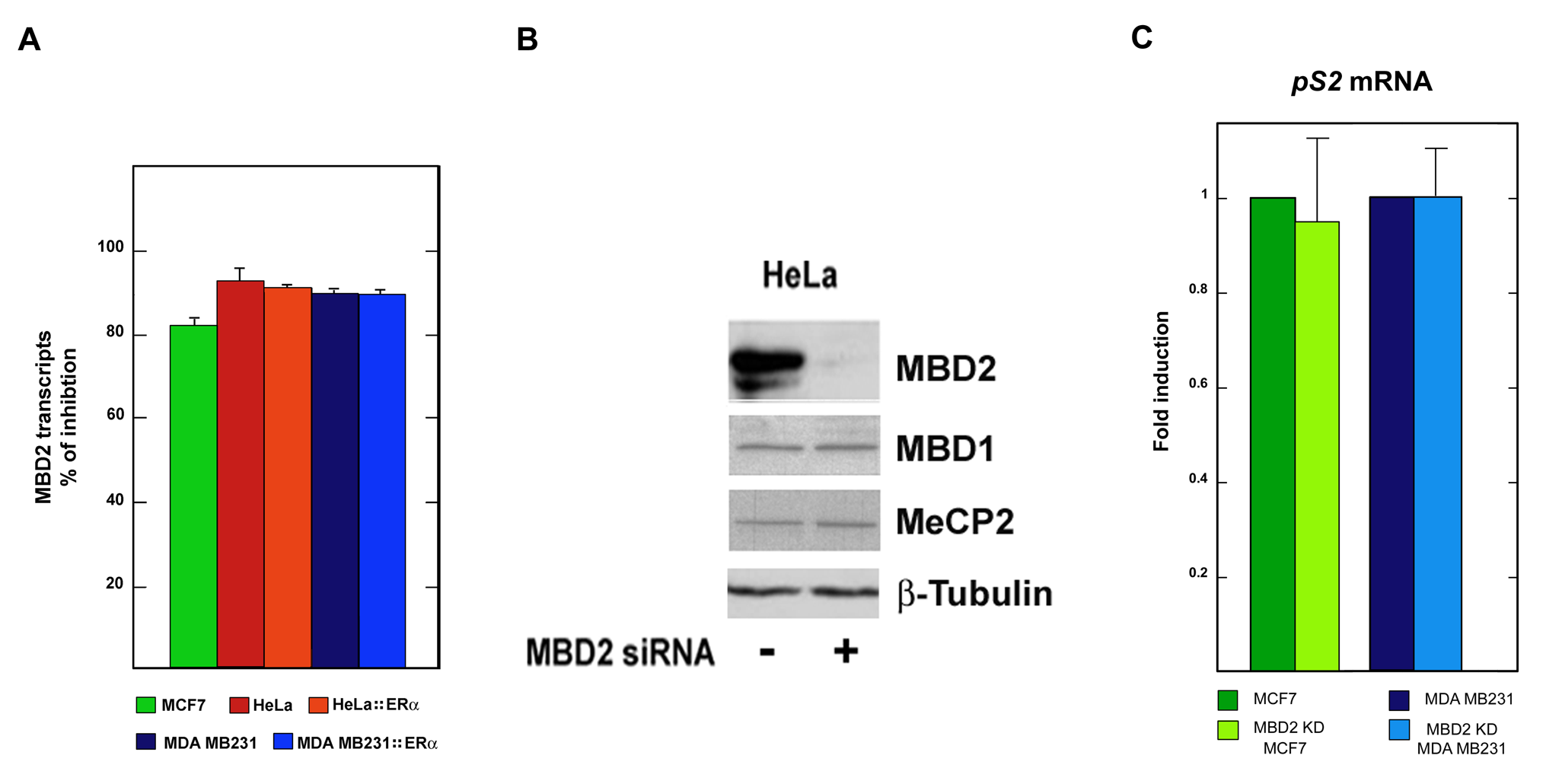

Supplement: Figure S1 — MBD2 siRNA treatments, supplementary data. (A) MBD2 expression in MBD2 siRNA transfected cells. Bar chart representing the efficiency of MBD2 siRNA in HeLa, MCF7 and MDA MB231 In mock treated cells, the initial amount of MBD2 molecules / µg of total RNA was: 7.4 106±1.3×10^6^, in HeLa cells; 5.8×10^6^ ±1×10^6^, in MDA MB231 cells and 3.2×10^6^±0.6×10^6^, in MCF7 cells. The efficiency of MBD2 siRNA was calculated from the MBD2 mRNA in treated cells compared with mock-treated cells. Each bar represents the mean ± standard deviation of, at least, three independent analyses. (B) MBD1, MBD2 and MeCP2 protein quantifications in HeLa cells expressing transient MBD2 siRNA. HeLa cells were pretreated for 72 h with MBD2 siRNA and again for 24 h. Mock-treated cells were transfected with a non-specific siRNA. Immunoblot analysis of MBD2, MBD1 and MeCP2 proteins in mock-treated and in MBD2 siRNA-treated HeLa cells. MBD2, MBD1 and MeCP2 proteins were probed using rabbit polyclonal antibodies. The same membrane was then stripped and probed using a mouse β-tubulin antibody as a loading control. (C) Bart chart showing the fold change of pS2 expression in MCF7 and MDA MB231 cells depleted in MBD2. pS2 transcripts were quantified by real-time RT-PCR. The fold change was calculated from the amount of pS2 mRNA in treated cells compared with mock-treated cells. Each bar represents the mean ± standard deviation of, at least, three independent analyses. (0.52 MB TIF) [file pone.0009665.s001.tif]
