# Supplementary material for: A Role for Methyl-CpG Binding Domain Protein 2 in the Modulation of the Estrogen Response of pS2/TFF1 Gene
Source: PLoS One. 2010 Mar 12;5(3):e9665. doi: 10.1371/journal.pone.0009665 (PMC2837351; doi:10.1371/journal.pone.0009665)
Supplement: Table S1 — List of primers. (0.03 MB DOC) [file pone.0009665.s002.doc]

**Table S1.** Primers used in the PCR and RT-PCR reactions

| **Name**  PCR bisulphite pS2 -464 to +67  PCR bisulphite pS2 +37 to +314  ChIP pS2 ERE  ChIP pS2 promoter  pS2 RT-PCR  pS2 RT-PCR Q  β-actin RT-PCR  β-actin RT-PCR Q  MBD2 RT-PCR | **Forward**  5'-AGA ATT AGT TTA GGT TTA GA-3'  5'-GGG TTT TGT TAT TTT GAG TTA-3'  5'-ATT AGC TTA GGC CTA GAC-3'  5'-CTA TAA AAT CCG GGG CTC GG -3'  5' GAG-AAC-AAG-GTG-ATC-TGC-GC 3'  5’ ATA-CCA-TCG-ACG-TCC-CTC-CA 3’  5' ACA-CTG-TGC-CCA-TCT-ACG-AGG 3'  5’ AGG-CCA-ACC-GCG-AGA-AGA-TGA 3’  5' TCA-GAA-GCA-AGC-CTC-AGT-TG 3' | **Reverse**  5'-AAT CAC CTT ATT CTC CAT AA-3'  5'-AAC AAT AAC CAC CAT AAA AAA C-3'  5'-CTG AGG GAT CTG AGA TTC A-3'  5'-CCC TGC CAC CCT GAG TTA CT -3'  5' TGG-TAT-TAG-GAT-AGA-AGC-ACC 3'  5’ AAG-CGT-GTC-TGA-GGT-GTC-CG 3’  5' AGG-GGC-CGG-ACT-CGT-CAT-ACT 3'  5’ GCC-GTG-GTG-GTG-AAG-CTG-TAG 3’  5' CAG-AGC-TTG-TGT-GCA-AAG-CA 3' |
| --- | --- | --- |

**Table S1.** *Chatagnon et al.*
